# Ruptured arterial aneurysm in Wegener’s granulomatosis: a case report

**DOI:** 10.1186/s13256-021-02955-7

**Published:** 2021-07-12

**Authors:** A. Gravos, K. Katsifa, P. Tselioti, V. Grammatikopoulou, K. Sakellaridis, S. Kanakaki, C. Tsapas, A. Destounis, H. Moschouris, I. Athanasiadou, F. Chatzivasiloglou, E. Ivanova, A. Prekates

**Affiliations:** 1 Intensive Care Unit (ICU), Tzaneio General Hospital of Piraeus, Dodonis 26, Kamatero, PC: 13451 Greece; 2 Radiology Department, Tzaneio General Hospital of Piraeus, Kamatero, Greece

**Keywords:** Granulomatosis with polyangiitis, Immunosuppression therapy, Aneurysm

## Abstract

**Background:**

Aneurysm formation is a possible, but rare, complication of granulomatosis with polyangiitis, known as Wegener’s granulomatosis. Urgent diagnosis and therapy is very important because a ruptured aneurysm could be life threatening.

**Case presentation:**

We, therefore, present the case of a 63-year-old Greek man who was diagnosed with granulomatosis with polyangiitis and retroperitoneal hematoma due to ruptured aneurysm in renal artery and upper pancreaticoduodenal artery. His clinical course was complicated by acute renal failure and acute respiratory failure due to alveolar hemorrhage. Emergency coil embolization was performed. Postembolization recovery was uneventful; no bleeding occurred. The patient underwent mechanical ventilation and continuous veno-venous hemofiltration and received combined immunosuppression and supportive therapy, but eventually died 30 days after admission to hospital from severe septic shock and multiple organ failure.

**Conclusion:**

Endovascular treatment is the therapy of choice, especially for patients with ruptured aneurysms that are hemodynamically stable. Early diagnosis is very important, as urgent embolization and early initiation of immunosuppression therapy are the treatment of choice.

## Background

Wegener’s granulomatosis, nowadays known as granulomatosis with polyangiitis (GPA), is a multisystemic disease characterized by granulomatous and systemic necrotizing vasculitis. Inflammation mainly affects the small-to-medium-sized arteries of the kidneys, upper airways, and the lungs [1].

Granulomatosis with polyangiitis (GPA) is an immune-mediated and complex disorder in which tissue injury results from the interaction of a highly specific immune response and an initiating inflammatory event. Part of this response is directed against previously shielded epitopes of neutrophil granule proteins, leading to autoantibodies known as antineutrophil cytoplasmic autoantibodies (ANCA) [2]. Approximately 85% of patients with GPA are ANCA positive. The most commonly identified and evaluated autoantibodies in GPA recognize the autoantigens proteinase 3 (PR3-ANCA), observed in 70–80% of patients, and myeloperoxidase (MPO-ANCA), observed in 10% of patients [3].

In the early stages of ANCA-associated vasculitis, endothelial cells may recruit inflammatory cells and enhance their adhesion to sites of vascular injury. This could lead to aneurysm formation in these arteries [4]. Formation of aneurysms is a very rare complication of GPA, and only a few cases [5-24] have been reported in the literature. We, therefore, present a case and therapeutic interventions of arterial aneurysms in GPA.

## Case presentation

### Patient information

A 63-year-old Greek man, active smoker, was admitted to the emergency department with a 2-month history of dyspnea and presented with acute abdominal pain. One month ago, he was diagnosed with GPA by lung biopsy and laboratory testing and was receiving low-dose dexamethasone (15 mg/day). His other medical and family history was unremarkable. He was not using anticoagulant agents.

### Clinical findings

On physical examination, his blood pressure was 110/70 mmHg, pulse rate 80 beats/minute, and respiration rate 20 breaths/minute, and he was afebrile. His height was 170 cm and weight 70 kg. His heart sounds were regular without any murmur, and his respiratory sounds were clear. He had abdominal dilatation with diffuse sensitivity to palpation and dullness on percussion. There were no inflamed joints, nasopharyngeal abnormalities, or dermatologic manifestations. On admission, there was no need for intubation and mechanical ventilation, and the patient was hemodynamically stable and with normal neurological examination.

### Diagnostic assessment

**Fig. 1.**
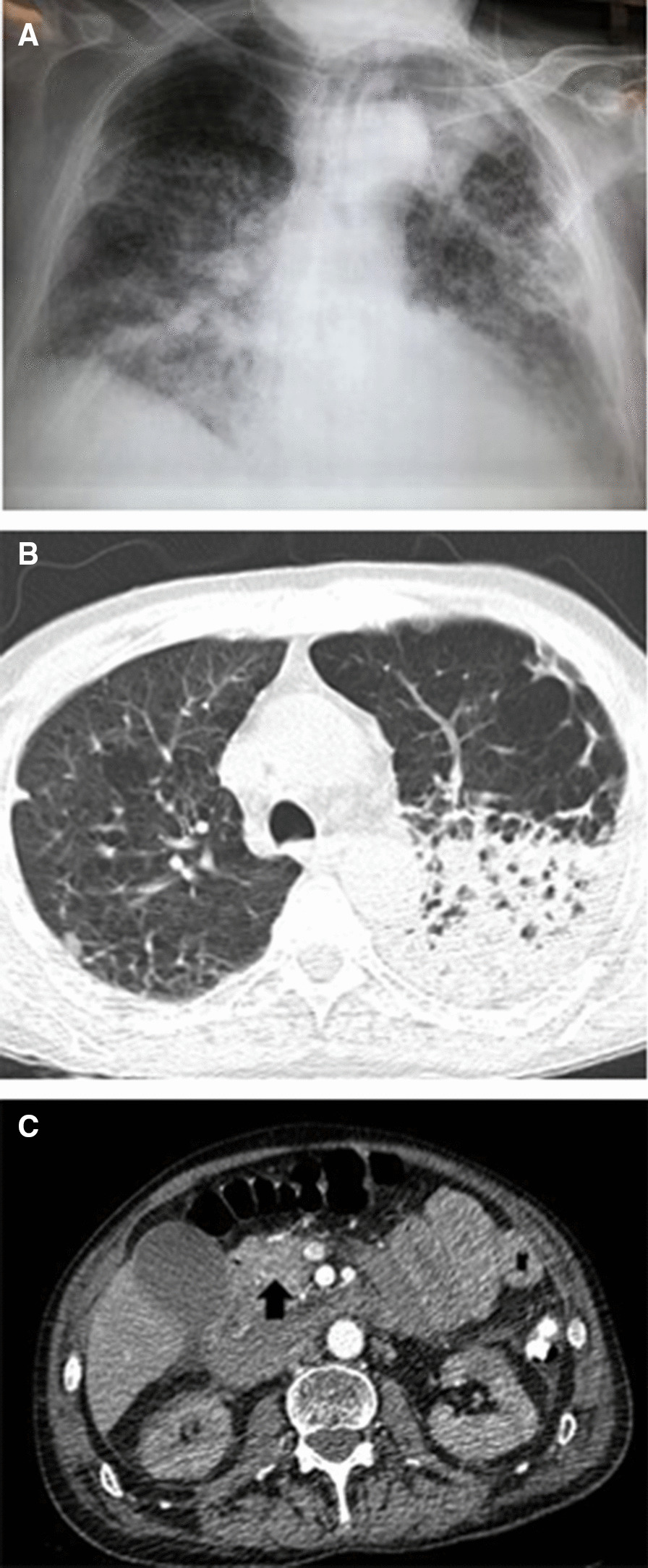
**a** CXR of the patient on admission showing infiltrative shadows in both lungs, especially in the left upper lobe. **b** Chest CT scan showing multiple intraparenchymal cavitated nodules in the left lung. **c** Abdominal CT scan showing a large retroperitoneal hematoma (arrow) and multiple aneurysms of the renal, hepatic, and pancreaticoduodenal arteries

Complete laboratory testing and blood gases were obtained, and urgent computed tomography (CT) scan of the lungs, brain, and upper and lower abdomen was performed. Chest X-ray (CXR) showed an infiltrative shadow in the upper left lobe (Fig. 1a), while CT scan showed multiple intraparenchymal cavitated nodules in the left lung (Fig. 1b) and a large retroperitoneal hematoma and multiple aneurysms of the renal, hepatic, and pancreaticoduodenal arteries (Fig. 1c). There were no other brain or chest aneurysms, as confirmed by whole-body CT angiography.

Laboratory tests showed: leukocyte count: 16 × 10^3^/μL (80% neutrophils, 10% lymphocytes, 5% monocytes, and 2% eosinophils); hemoglobin 7.2 g/dL; hematocrit 21.1%; platelet count 235 × 10^3^/μL; C-reactive protein 46 mg/dL; urea 236 mg/dL; serum creatinine (SCr) 7.6 mg/dL (2 months prior to admission, his SCr level was 0.7 mg/dL); and albumin: 2.3 g/dL. His liver enzyme levels and electrolytes were normal. His erythrocyte sedimentation rate (ESR) was 122 mm/hour. Urinalysis showed protein of 2+ and blood of 2+, but without red blood cell casts or dysmorphic red blood cells. Proteinase-3 antineutrophil cytoplasmic antibody (PR3-ANCA), c-ANCA, and antinuclear antibody were positive, while antiglomerular basement antibody (GBM) and myeloperoxidase antineutrophil cytoplasmic antibody (MPO) were negative. The serum total complement and complement 3 and 4 levels and serum protein electrophoresis were within normal limits. Viral serology for human immunodeficiency virus (HIV), hepatitis B virus, and hepatitis C virus was normal.

### Therapeutic interventions

**Fig. 2.**
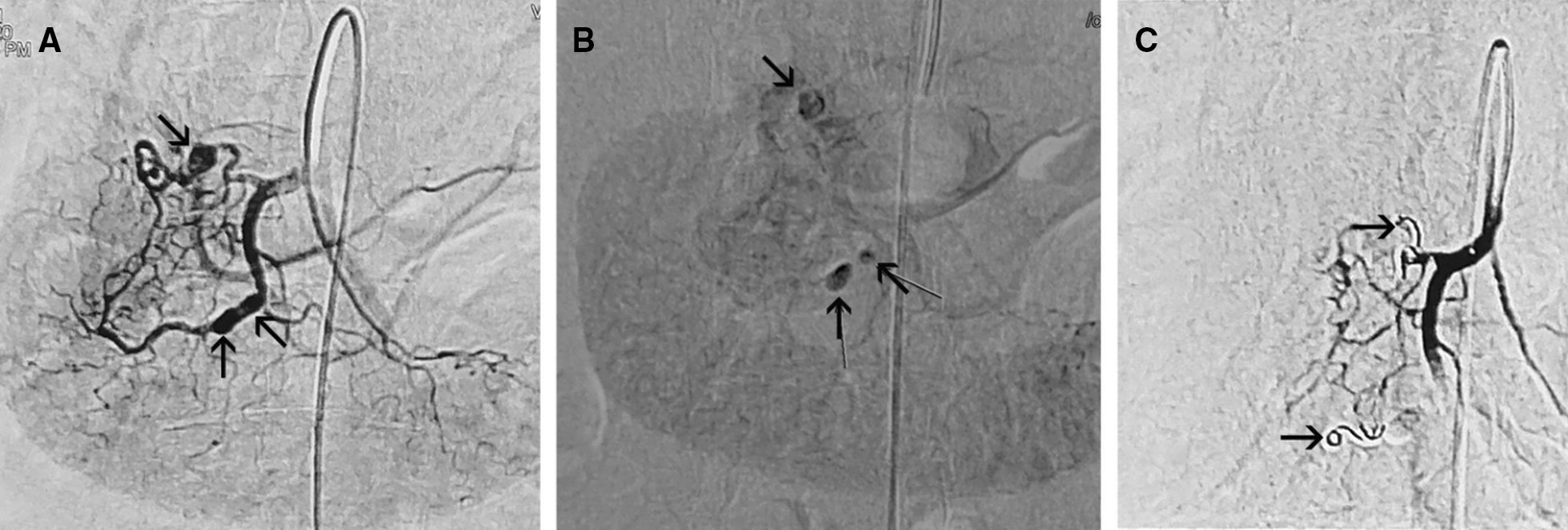
Digital subtraction angiograms (DSAs, **A** arterial, **B** parenchymal phase) showing three pseudoaneurysms (arrows) of the pancreaticoduodenal arteries. DSA postembolization (**C**), with microcoils (arrows) and Gelfoam shows obliteration of the pseudoaneurysms

Automatic rupture of these aneurysms with concomitant retroperitoneal hematoma was suspected, and urgent angiography of the celiac artery and renal arteries was performed. Angiography showed multiple aneurysms of the hepatic, renal, and pancreaticoduodenal arteries and embolization of the pancreaticoduodenal arteries was performed, as these arteries were the suspected cause of the hematoma (Fig. 2).

The following day of embolization, the patient deteriorated and was intubated because of acute respiratory failure and was transferred to the ICU. His respiratory failure was attributed to alveolar hemorrhage, as confirmed by bronchoscopy and bronchoalveolar lavage. He did not develop pancreatitis after the embolization. In the ICU, the patient was hemodynamically stable, on mechanical ventilation, and sedated. His blood gases improved immediately after intubation. Due to acute renal failure, he was placed in continuous veno-venous hemofiltration (CVVHDF) and received pulse therapy with methylprednisolone 1 g/day for 3 days followed by maintenance therapy with prednisolone 50 mg/day and 700 mg cyclophosphamide pulses, after proper hydration, two times on a 2-week basis. The daily dose of prednisone was tapered gradually.

### Follow-up and outcomes

The patient deteriorated gradually, despite the initial improvement and the early and combined immunosuppression and supportive therapy. He was subjected to tracheostomy on the 15th day of admission, but developed severe thrombocytopenia and neutropenia, attributed to cyclophosphamide. Blood cultures on the 20th day revealed *Acinetobacter baumannii*, for which he received combined antibiotic therapy. His clinical and hemodynamic condition and results of laboratory and blood gas testing deteriorated gradually and finally died on the 30^th^ day of admission from severe septic shock and multiple organ failure.

## Discussion

In January 2011, the Boards of Directors of the American College of Rheumatology (ACR), the American Society of Nephrology (ASN), and the European League Against Rheumatism (EULAR) recommended that the name “Wegener’s granulomatosis” be changed to “granulomatosis with polyangiitis,” abbreviated as GPA. This change is a plan to gradually shift from honorific eponyms to a disease-descriptive or etiology-based nomenclature. Antineutrophil cytoplasmic autoantibody (ANCA)-associated vasculitis (AAV) includes microscopic polyangiitis (MPA), renal-limited vasculitis (RLV), eosinophilic granulomatosis with polyangiitis (EGPA, Churg-Strauss), and granulomatosis with polyangiitis (GPA). All are associated with ANCA and have similar features on renal histology [25, 26].

GPA is a multisystemic, but rare, disease characterized by granulomatous and systemic necrotizing vasculitis. The expression of glycoprotein enzymes, such as PR3 on the surface of cytokine-primed neutrophils, is the key to the pathogenesis of GPA. PR3-ANCA then binds to this antigen and activates neutrophils. This results in cytotoxicity to vascular endothelial cells via release of proinflammatory cytokines and lytic enzymes. GPA affects mainly the medium and small arteries of the kidneys and the respiratory tract [27, 28]. Inflammation can also lead to aneurysm formation in larger arteries, but aneurysm formation is a very unusual complication. About 20 cases [5-24] are described with large-vessel aneurysms in patients with GPA.

A review in 2017 [5] revealed that arterial aneurysms were diagnosed within 1 month from disease onset and were observed more commonly in men. Most patients had symptoms of aortitis and ischemia, such as abdominal and back pain. Most cases were large-vessel aneurysms, medium-vessel aneurysms, branches of the celiac axis, and branches of the renal artery. Rupture is not a rare complication, requiring urgent recognition and treatment either with embolization or surgery [5].

Treatment involves the combination of endovascular and surgical interventions with immunosuppressive agents such as high-dose methylprednisolone, cyclophosphamide, and/or rituximab to prevent aneurysm rupture and control of hemorrhage [29, 30]. Our patient was treated by endovascular intervention combined with high-dose methylprednisolone and cyclophosphamide, but finally died due to septic shock from *Acinetobacter baumannii*.

Initial immunosuppression therapy in granulomatosis with polyangiitis (GPA) typically consists of high-dose glucocorticoids combined with either cyclophosphamide or rituximab. Some studies have described the use of both cyclophosphamide and rituximab for initial therapy (rather than using one of these two agents), although this approach is controversial. Some patients with severe disease may benefit from the addition of plasma exchange, especially those with concurrent anti-GBM autoantibody disease and pulmonary hemorrhage [31].

The mortality rate in untreated generalized GPA is about 90% at 2 years, usually due to respiratory or renal failure. Thus, the use of aggressive initial immunosuppression is justified. The introduction of initial therapy with cyclophosphamide and glucocorticoids has markedly diminished the mortality [32].

## Conclusions

We present a rare case of a life-threatening ruptured aneurysm in a patient with GPA. Clinicians should be aware of this rare complication of GPA, which can be the cause of retroperitoneal hematoma. Accurate diagnosis in combination with early immunosuppression and embolization therapy should be performed. Endovascular intervention is the treatment of choice, accompanied by surgical therapy if embolization fails or is not available.

## Data Availability

Data supporting our findings can be found in the patient’s electronic file.

## Abbreviations

ICU: Intensive care unit
GPA: Granulomatosis with polyangiitis
ANCA: Antineutrophil cytoplasmic autoantibodies
PR3-ANCA: Proteinase 3 antineutrophil cytoplasmic autoantibodies
MPO-ANCA: Myeloperoxidase antineutrophil cytoplasmic autoantibodies
CT: Computed tomography
CXR: Chest X-ray
ESR: Erythrocyte sedimentation rate
GBM: Antiglomerular basement antibody
CVVHDF: Continuous veno-venous hemofiltration
ACR: American College of Rheumatology
ASN: American Society of Nephrology
EULAR: European League Against Rheumatism
AAV: ANCA-associated vasculitis
MPA: Microscopic polyangiitis
RLV: Renal-limited vasculitis
EGPA: Eosinophilic granulomatosis with polyangiitis
